# Effect of acupuncture on gut microbiota in participants with subjective cognitive decline: Retraction

**DOI:** 10.1097/MD.0000000000030340

**Published:** 2022-08-05

The article, “Effect of acupuncture on gut microbiota in participants with subjective cognitive decline”,^[1]^ which appears in Volume 101, Issue 18 of *Medicine*, is being retracted due to improper authorship, the use of data without appropriate consent, and omitting the citation of Yan, Zhou, Wang, et al^[2]^ in the following relevant sections: inclusion and exclusion criteria, intervention methods, clinical outcome indicators, and Figure 1. The authors agreed to retract this article.
